# Crystal structure of an intramembranal phosphatase central to bacterial cell-wall peptidoglycan biosynthesis and lipid recycling

**DOI:** 10.1038/s41467-018-03547-8

**Published:** 2018-03-20

**Authors:** Sean D. Workman, Liam J. Worrall, Natalie C. J. Strynadka

**Affiliations:** Department of Biochemistry and Molecular Biology and the Center for Blood Research, University of British Columbia, 2350 Health Sciences Mall, Vancouver, BC V6T 1Z3 Canada

## Abstract

Undecaprenyl pyrophosphate phosphatase (UppP) is an integral membrane protein that recycles the lipid carrier essential to the ongoing biosynthesis of the bacterial cell wall. Individual building blocks of peptidoglycan are assembled in the cytoplasm on undecaprenyl phosphate (C55-P) before being flipped to the periplasmic face, where they are polymerized and transferred to the existing cell wall sacculus, resulting in the side product undecaprenyl pyrophosphate (C55-PP). Interruption of UppP’s regeneration of C55-P from C55-PP leads to the buildup of cell wall intermediates and cell lysis. We present the crystal structure of UppP from *Escherichia coli* at 2.0 Å resolution, which reveals the mechanistic basis for intramembranal phosphatase action and substrate specificity using an inverted topology repeat. In addition, the observation of key structural motifs common to a variety of cross membrane transporters hints at a potential flippase function in the specific relocalization of the C55-P product back to the cytosolic space.

## Abstract (web summary)

Undecaprenyl pyrophosphate phosphatase (UppP) recycles the lipid carrier essential for bacterial cell wall synthesis. Here authors present the crystal structure of UppP from *E. coli* at 2.0 Å resolution, which sheds light on its phosphatase mechanism and indicates a potential flippase role for UppP.

## Introduction

The translocation of sugars and glycan chains across membranes using long poly-prenyl phosphate lipids is a process that is highly conserved across all kingdoms of life. In bacteria, the most common carrier lipid is undecaprenyl phosphate (C55-P). As well as playing a role in protein glycosylation, C55-P acts as the universal carrier lipid in the biosynthesis of peptidoglycan, wall teichoic acids, and many other major bacterial cell wall polymers^1^. During this process, nucleotide-activated sugar moieties are transferred to C55-P at the cytoplasmic face of the membrane bilayer. The resulting glycolipids are subsequently flipped to the periplasmic face of the plasma membrane by specialized glycolipid flippases such as MurJ and TagGH^2, 3^. The lipid-activated glycan moieties are then covalently transferred to specific glycan acceptors in the periplasmic space, in most cases resulting in the release of undecaprenyl pyrophosphate (C55-PP) as a byproduct^1, 4^. In addition to being released as a byproduct of cell wall biosynthesis, C55-PP is synthesized de novo by the cytosolic enzyme undecaprenyl pyrophosphate synthase (UppS). In both cases, C55-PP must be dephosphorylated to C55-P before it can be linked to a sugar or glycan. Disruption of the biosynthesis or recycling of C55-PP halts peptidoglycan biosynthesis and subsequently results in cell lysis. While the synthesis of C55-PP by UppS has been well characterized^5^, its essential dephosphorylation to C55-P remains poorly understood, as does the mechanism by which C55-P would translocate from the periplasmic to cytoplasmic leaflet of the plasma membrane.

A 30 kDa polytopic integral membrane protein, undecaprenyl pyrophosphate phosphatase (UppP; also referred to in previous literature as BacA), was first identified in a screen for genes that could confer resistance to the antibiotic bacitracin upon amplification^6^. While it was first proposed to function as an undecaprenol kinase, it has since been shown that UppP is rather a C55-PP phosphatase^7^. Knockout of *uppP* in *Escherichia coli* resulted in a 75% decrease in C55-PP phosphatase activity, with little observed effect on apparent in vitro growth^7^. A later study identified two phosphatidic acid phosphatase 2 (PAP2) family proteins, PgpB and YbjG, as the enzymes responsible for the residual C55-PP phosphatase activity with a *uppP*/*pgpB*/*ybjG* knockout lethal^8^. Despite the apparent redundancy in vitro, effects of *uppP* knockouts in vivo are significant, with, for example, deficient *Staphylococcus aureus* and *Streptococcus pneumoniae* showing attenuated virulence in mouse models of infection^9^ and *uppP*-deficient *Mycobacterium tuberculosis* showing impaired biofilm formation^10^. These results, combined with the historical success of antibiotics targeting peptidoglycan biosynthesis^11^, suggest that UppP could be a viable target for the development of therapeutics.

Bioinformatic and biochemical analyses of *E. coli* UppP (*Ec*UppP) have identified two conserved motifs containing residues that are implicated in the phosphatase activity of the protein; however, there is no consensus on the identity of *Ec*UppP’s catalytic players, the mechanistic details of the dephosphorylation reaction, or on which face of the membrane dephosphorylation of C55-PP occurs. Site-directed mutagenesis studies of *Ec*UppP carried out by Chang et al. and by Manat et al. proposed, alternatively, His30 or Ser27 as the central catalytic residue involved^12, 13^. Both of these studies demonstrated that phosphatase activity was dependent on a divalent cation with a marked preference for magnesium and calcium^12, 13^. Topology mapping of PgpB and YbjG, the primary sequences of which are completely distinct from UppP, suggested that the catalytic motif of the PAP2 phosphatases would be directed toward the periplasmic face of the bilayer, and confirmation of PgpB’s topology was given by its recently determined X-ray crystal structures from both *E. coli* and *Bacillus subtilis*^14–16^. The results of these studies suggested that the PAP2 phosphatases could act as recycling enzymes at the periplasmic face of the inner membrane, with UppP carrying out dephosphorylation during de novo synthesis of the carrier lipid in the cytoplasm. However, a recent topology analysis of *Ec*UppP has suggested both of the proposed catalytic residues are also directed toward the periplasm^12^, again raising questions as to how cytoplasmic dephosphorylation during de novo synthesis might occur. Furthermore, while the specific glycolipid flippases involved in numerous biosynthetic pathways have been identified, and in some cases structurally characterized^17, 18^, there remains little information regarding the translocation of C55-P to the cytoplasmic face of the inner membrane^1^.

Here, we present the X-ray crystallographic structure of *Ec*UppP at 2.0 Å resolution. Our high-resolution structure reveals a surprising membrane topology and overall architecture predominantly found in ion channels and transporters, gives insight into the active site and mechanistic details allowing for the observed intramembranal C55-PP dephosphorylation and provides support for a potential role as a phosphatase-activated C55-P-specific flippase.

### Results X-ray crystallographic structure of *Escherichia coli* UppP

In order to identify suitable candidates for our structural studies, we screened a large number of bacterial UppP orthologues and sequence constructs for expression, detergent solubilization, stability, and monodispersity using fluorescence size exclusion chromatography^19^; full-length *Ec*UppP (residues 1–273) was a top selection for further structural studies. Activity of our recombinant *Ec*UppP was determined using a coupled phosphatase assay with resulting kinetics consistent with those previously published^12, 13^ (Supplementary Fig. 1a). Crystals were obtained using the lipid cubic phase (LCP) method^20^ but we note the crystals used for the ultimate structure determination were grown in crystallization conditions (100 mM NaCitrate pH 4, 150 mM NaCl, 300 mM LiSO4, 50% PEG 400) that promoted formation of the sponge phase. The crystals belonged to space group *C*222 with unit cell dimensions 109.88 × 146.08 × 40.19 Å and one molecule in the asymmetric unit (53.2% solvent content^21, 22^). Experimental phases were determined via single-wavelength anomalous dispersion (SAD) from ethyl mercury phosphate derivatized crystals. All residues could be readily traced in the resultant electron density maps with the exception of Met1-Asp3. Several monoolein lipid molecules of varying order and occupancy were also modeled and included in the refinement. The final refinement statistics to 2.0 Å resolution and stereochemical indicators for the resulting model are described in the “Methods” and shown in Table 1.

**Table 1:** Data collection and refinement statistics

	EtHgPO4 UppP	Native UppP
*Data collection*		
Space group	*C*222	*C*222
Cell dimensions		
* a*, *b*, *c* (Å)	111.19, 146.84, 40.20	110.05, 146.19, 40.23
α, β, γ (°)	90, 90, 90	90, 90, 90
Wavelength	1.0057	0.9795
Resolution (Å)	40.20–3.00 (3.11–3.00)	44.56–2.00 (2.07–2.00)
*R*_sym_ or *R*_merge_	0.1663 (0.942)	0.1083 (1.434)
*I*/σ*I*	14.38 (2.91)	8.11 (1.25)
Completeness (%)	99.70 (99.28)	95.58 (69.75)
Redundancy	12.1 (11.9)	5.7 (4.0)
*Refinement*		
Resolution (Å)		44.56–2.00 (2.07–2.00)
No. reflections		21,713
*R*_work_/*R*_free_		0.22/0.25
No. atoms		
Protein		2080
Ligand/ion		145
Water		44
*B*-factors		
Protein		44.78
Ligand/ion		67.20
Water		41.54
R.m.s deviations		
Bond lengths (Å)		0.006
Bond angles (°)		1.02

Values in parentheses are for highest-resolution shell

**Fig. 1:**
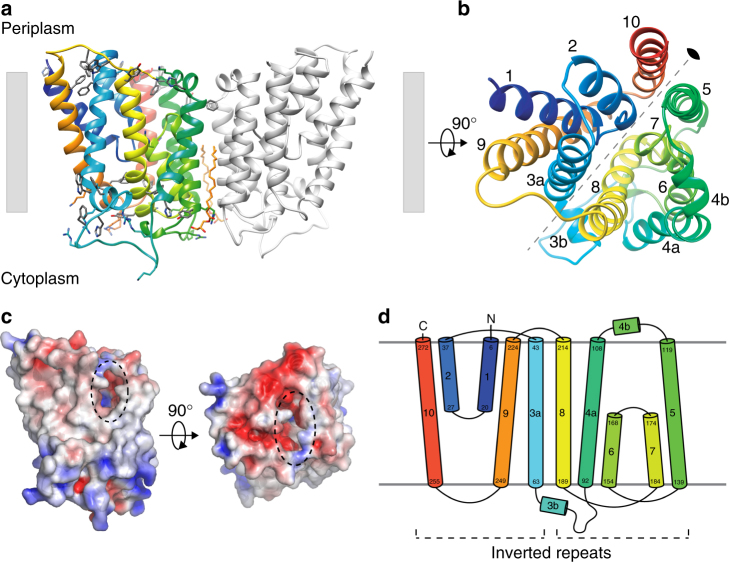
The crystal structure of *Ec*UppP at 2.0 Å resolution. **a** Cartoon representation of *Ec*UppP dimer with one monomer-colored rainbow from the N- (blue) to C-terminal (red). Aromatic and positively charged residues at the membrane interface shown as stick and highlight orientation with respect to the inner membrane (shown as gray bars). Two monoolein lipids at the dimer interface shown as stick. **b** Ninety-degree rotation from **a** viewed from periplasm. Colored as **a** and helices numbered. Twofold pseudosymmetry axis parallel to the plane of the membrane shown as gray dotted line. **c** Electrostatic surface potential of UppP monomer. Orientations as in **a** and **b**, respectively. Dotted circles indicate location of periplasmic substrate-binding cleft. **d** Secondary structure topology highlighting interlocked inverted repeat

Our refined structure reveals that *Ec*UppP is composed largely of ten membrane-embedded α-helices (see Fig. 1), contrary to previous predictions that it would contain between seven or eight^12, 13, 23^. Six helices are full-span transmembrane (TM) helices (α3–α5, α8–α10), while the remaining four make up two antiparallel reentrant helix-loop-helix regions (α1–α2, α6–α7) that are arranged in an inverted manner at what we propose is the enzyme active site and contain highly conserved residues that have been previously shown by mutagenesis to be crucial for the phosphatase activity of the protein^12, 13^. We observe a classic girdle of aromatic residues at the two leaflet interfaces, defining the membrane span (Fig. 1a), and analysis of surface properties suggests the orientation with respect to the lipid bilayer. Surface electrostatics illustrate a charge polarity with the hydrophilic surface defined by loops connecting helices α3a–α4 (encompassing helix α3b), α5–α6, α7–α8, and α9–α10 largely electropositive and predicted to be oriented to the cytoplasm with the extended loops α3a–α4 (Gly73–Thr91) and α5–α6 (Lys140–Thr153) harboring a large number of positively charged residues (Fig. 1a, c) in keeping with the positive inside rule of membrane protein topology^24^. Conversely, the surface containing the N and C termini along with loops connecting helices α2–α3, α4a–α5 (encompassing helix α4b) and α8–α9 project to the periplasm and form a predominantly electronegative surface (Fig. 1c).

Although *Ec*UppP crystallized with one molecule in the asymmetric unit, analysis of the crystal packing shows a crystallographic dimer with a twofold symmetry axis perpendicular to the plane of the membrane (Fig. 1a). Analysis with PISA^25^ shows an interface surface area of 973.5 Å^2^, which along with the parallel arrangement with respect to the membrane bilayer suggests it may be of physiological relevance. Gel filtration analysis demonstrates that detergent-solubilized *Ec*UppP elutes with a higher hydrodynamic radius and apparent molecular weight than theoretically expected for a monomer plus detergent micelle (Supplementary Fig. 1b). To further study this, we conducted chemical crosslinking using amine reactive cross-linkers disuccinimidyl suberate and ethylene glycol bis(succinimidyl succinate) and observed the accumulation of a dimer species (Supplementary Fig. 1b). Two crystallographically related lipids, modeled here as monoolein and well ordered in their entirety, are observed flanking the interface from the cytoplasmic surface (Fig. 1a), which could play a role in stabilization of the dimeric form, a reoccurring general feature recently shown to be of importance in membrane protein oligomeric interfaces^26^. Beyond this likely stabilization and the observed parallel disposition of the two monomers relative to the membrane leaflets, it is not evident from our structural data if UppP dimerization is also necessary for an additional allosteric (distinct active sites ~35 Å distance apart) or other functional role.

Intriguingly, analysis of the helical packing arrangement reveals *Ec*UppP has internal pseudosymmetry within each monomer, with a twofold rotation axis parallel to and bisecting the membrane midplane, which relates a five-helix motif encompassing respective reentrant regions (see Fig. 1b, d). The contiguous second repeating domain (helices α4–α8) is inserted in sequence between helices α3 and α9 of the first repeat, consequently resulting in an interlocked inverted domain-swap-like arrangement, where the C-terminal helices of the first repeat are separated in primary structure but tightly associated in tertiary structure. Superposition of the domains reveals a strong degree of structural similarity with a backbone RMSD of ~3.2 Å (Supplementary Fig. 2a) and a calculated sequence similarity for the structurally aligned regions of 29% (identity 19%), suggesting a common evolutionary origin. Inverted twofold pseudosymmetry is common among α-helical transporters, receptors, and channels^27^. Suggestively, this interlocked inverted topology repeat has primarily been described in proteins involved in secondary transport across lipid bilayers^27–29^ and the functional implications of this are discussed below.

### The substrate-binding pocket and active site

**Fig. 2:**
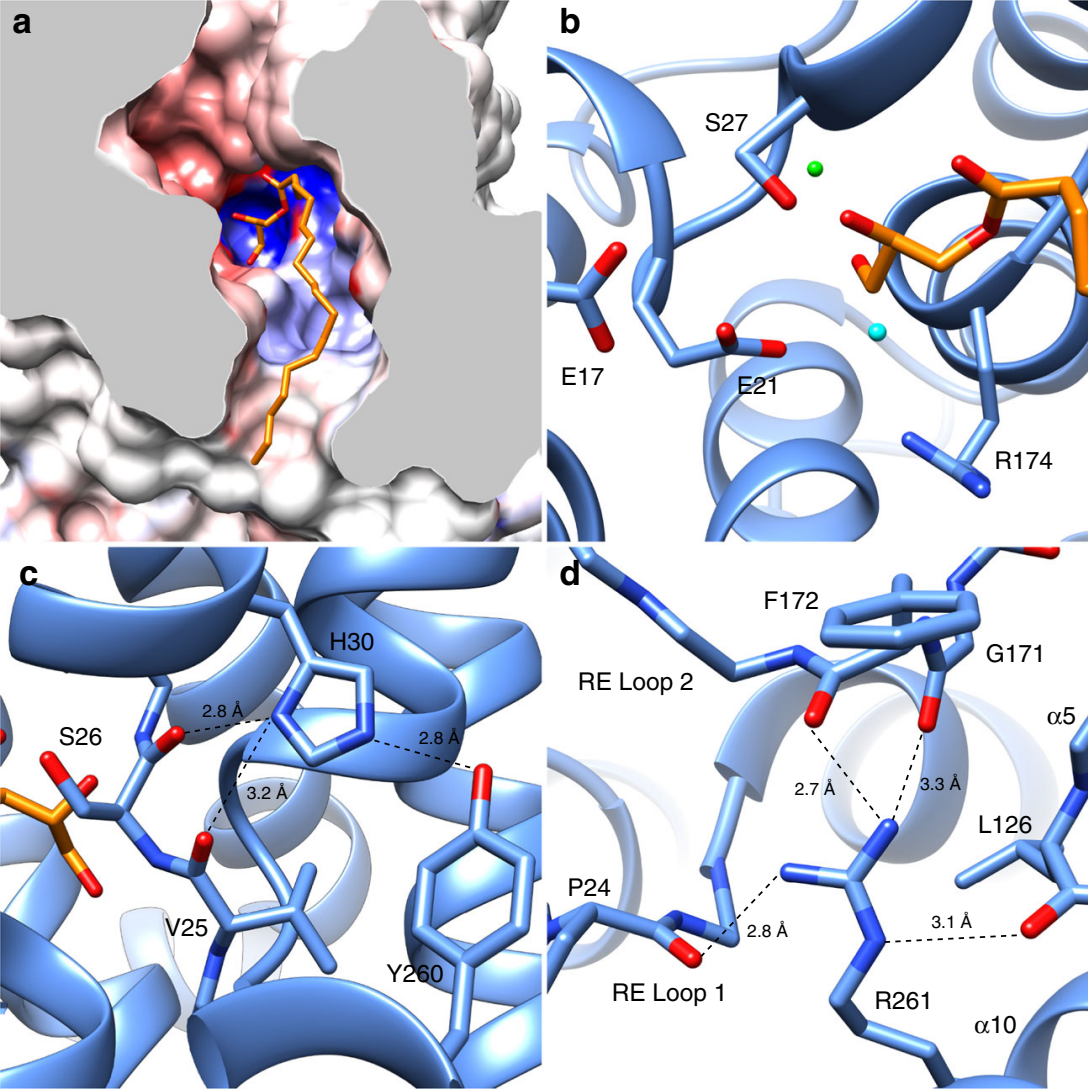
The *Ec*UppP substrate-binding pocket. **a** Clipped view of the *Ec*UppP substrate-binding pocket from a periplasmic viewpoint showing the electronegative funnel and deep hydrophobic channel leading into the positively charge basin formed by the antiparallel inverted reentrant helices defining the active site. An observed monoolein lipid is shown in stick with the polar headgroup bound in the active site pocket and lipid tail exiting the cleft along the hydrophobic channel. **b** Magnified view of the *Ec*UppP active site with key catalytic residues shown in stick. Two modeled active site waters are shown as cyan and green spheres representing the proposed catalytic water and a putative cation-binding site, respectively. **c** His30 forms structural hydrogen bonds with the backbones amides of Val25 and Ser26, as well as the hydroxyl oxygen of Tyr260. **d** Arg261 is buried at the membrane midplane (also see Supplementary Fig. 6) and forms a hydrogen-bonding network that links both reentrant loops (RE Loop 1/2) through the backbone amides of Pro24, Gly171, and F172, in addition to α5 through the backbone amide of Leu126

**Fig. 3:**
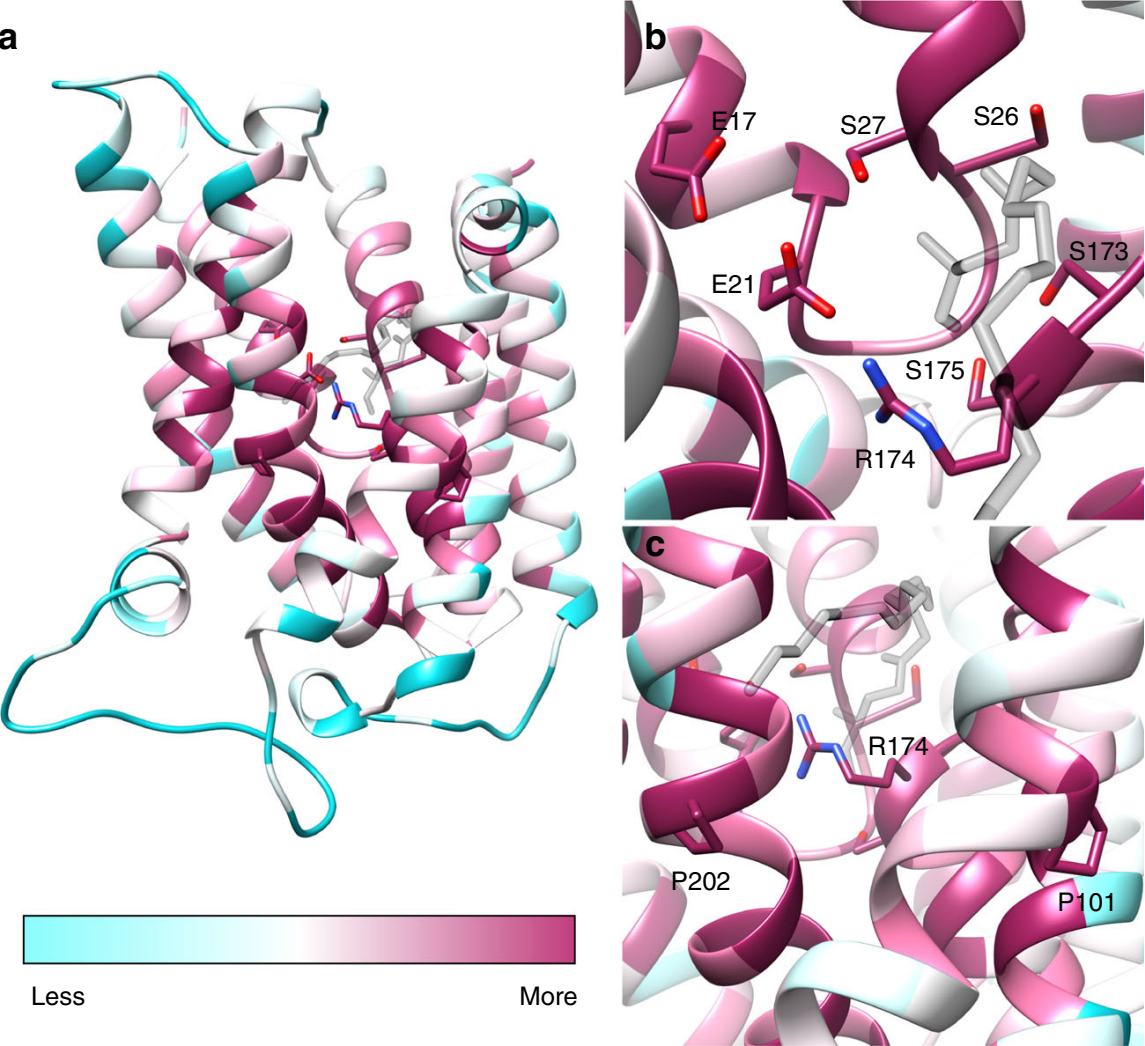
Conservation of *Ec*UppP catalytic core. **a** *Ec*UppP structure colored according to sequence conservation from low (cyan) to high (maroon). Highly conserved residues cluster near the catalytic core of the protein. Monoolein displayed in transparent gray. **b** Top-down view of the active site, showing the highly conserved nature of residues implicated in catalysis (E17, E21, S27, and R174) and maintenance of the reentrant loop architecture (S26, S173, and S175). **c** Magnification of strictly conserved proline residues that facilitate the bending of α4a and α8 to create the cleft that gives access to the active site

A prominent feature of the *Ec*UppP structure is the large cleft open to both the aqueous periplasm and the hydrophobic acyl core of the bilayer. Analysis with the 3 V server^30^ calculates a cleft volume of 1914 Å^3^ with approximate dimensions of 18 Å (l) × 8 Å (w) × 18 Å (h) (Fig. 2a and Supplementary Fig. 3a). Electrostatic surface analysis shows a negatively charged funnel at the periplasmic face feeding into a deep hydrophobic channel which in turn empties into an electropositive basin at the pocket formed by the inverted reentrant helices (Figs 1c and 2a). The V-shaped opening to the lipid environment of the periplasmic leaflet is framed by helices α4a and α8, which are both kinked at invariant prolines (Pro101 and Pro202) positioned at the midplane of the membrane (Fig. 3a and Supplementary Fig. 3a). Many of the residues lining the cleft are well conserved (Fig. 3); previous bioinformatic and biochemical analysis identified two highly conserved sequence motifs important for function, which we show map to the loop regions connecting the respective reentrant helices (residues 17–30 and 160–179; Supplementary Fig. 4) situated at the membrane midplane and which structurally define the active site. The location is in notable contrast to the surface localized active sites previously characterized in the structurally and functionally distinct PAP2 and other integral membrane spanning phosphatases^15, 16^. Electron density for a single monoolein lipid is observed in the cleft with the well-ordered glycerol headgroup bound in the deep electropositive basin near a number of highly conserved residues we believe to be central to phosphatase action and with its more dynamic lipid tail oriented along the hydrophobic channel and exiting at the base of the opening to the membrane hydrophobic core (Fig. 2a and Supplementary Fig. 3b, c). Like C55-PP, monoolein is a single chain lipid with a hydrophilic headgroup and we propose the observed binding orientation is representative of the native substrate complex.

The reentrant loops are similar in both sequence and structure (Supplementary Fig. 2b, c). Strictly conserved proline residues (Pro24 and Pro170) break the respective N-terminal helices (α1 and α6) and, coupled with the more polar characteristics of the proximal residues contribute to the reentrant conformation, while conserved serine residues (Ser26 and Ser173) form the N-terminal cap of the C-terminal helices (α2 and α7) providing interactions with the peptide backbone that serve to orient the key catalytic residues (Ser27 and Arg174; discussed below), which are conserved in alignment (Supplementary Fig. 2b, c). Preceding biochemical data involving mutation of a number of conserved, titratable residues have been shown to result in substantial decreases in phosphatase activity in *Ec*UppP^12, 13^ and the majority of these map to these structural motifs and surrounding residues (Supplementary Fig. 5).

The first reentrant helix-loop-helix contains conserved residues Glu17, Glu21, Ser26, Ser27, and His30, which were previously targeted for mutation^12, 13^. Notably, separate studies proposed either Ser27^13^ or His30^12^ as the central nucleophile involved. From our structure, Ser27 unambiguously fulfills this role with its side chain projecting from the N-terminal end of helix α2 into the active site, where it forms a strong hydrogen bond (2.7 Å) with the secondary alcohol of the bound monoolein glycerol headgroup (Fig. 2b). Our experimental data supports this with mutation of Ser27 to alanine completely abrogating activity (Supplementary Fig. 1a). The carboxylate side chains of both Glu17 and Glu21 are located directly proximal to Ser27, with Glu21 forming a direct hydrogen bond to its side chain hydroxyl that suggests a role in catalysis. His30, on the other hand, lies on the opposite side of helix α2, away from the active site, and appears to play a purely structural role (Fig. 2c and Supplementary Fig. 5c), albeit one central to the optimal positioning of Ser27. The side chain imidazole forms hydrogen bonds with the carbonyl oxygens of Val25 and Ser26 (Fig. 2c). These hydrogen bonds serve to stabilize the 3_10_ nature in this N-terminal region of an otherwise classic α-helix that is critical for appropriately orienting Ser27 into the substrate-binding pocket. His30 further forms a strong hydrogen bond (2.8 Å) with the side chain hydroxyl oxygen of Tyr260, providing structural stabilization between helices α2 and α10 (Fig. 2c). Thus, the decreased phosphatase activity observed for His30Ala (Supplementary Fig. 1a) is presumably due to destabilization from the loss of these key structural interactions, as well as the consequent effect on the sub-optimal positioning of the first reentrant loop in the active site. Both Tyr260 and the neighboring Arg261, mutation of which completely abrogates activity^12^, are also very highly conserved (Supplementary Fig. 4) and form multiple noncovalent interactions that further serve to orient both reentrant active site loops (Fig. 2c, d). The side chain guanadinium of Arg261 is remarkably fully buried within a largely hydrophobic interface at the juxtaposition of helices α2, α5, α7, and α10, where it forms a hydrogen bond network with both reentrant loops at the carbonyls of the invariant Pro24 and Gly171 as well as with the adjacent residue (Phe172 in *Ec*UppP) and the backbone carbonyl of Leu126 on helix α5 (Fig. 2d and Supplementary Fig. 6).

The second reentrant loop harbors a highly conserved Ser-Arg-Ser motif (Supplementary Fig. 2b, c). Remote homology detection with Phyre2^31^ identifies similarity to the P-loop motif found in dual specificity phosphatases (DUSPs)^32–34^, where the role of the conserved arginine is coordination and electrostatic polarization of the phosphate moiety of a phosphothreonine or phosphotyrosine allowing a cysteine nucleophile (located at N-6 in the P-loop and not conserved in UppP), to attack and optimally promote subsequent hydrolysis and phosphate release^32–34^. In *Ec*UppP, Arg174 projects into the active site pocket in close proximity to the monoolein headgroup (Fig. 2b) and is suitably positioned to fulfill the analogous role in cleavage of the C55 pyrophosphate (see below). Ser173 is the N-terminal capping residue for helix α7 with its side chain hydroxyl forming a hydrogen bond with the backbone carbonyl of Gly176. Mutation of Ser173 and Arg174 to alanine reduced activity ~50-fold and ~1000-fold, respectively^13^.

In addition to the interactions noted above, it appears that the antiparallel orientation of the four reentrant helices is partially maintained by structural waters, clearly defined in our 2.0 Å resolution maps, that are coordinated by the backbone amides of the residues making up the loop linking the reentrant helices.

## Discussion

**Fig. 4:**
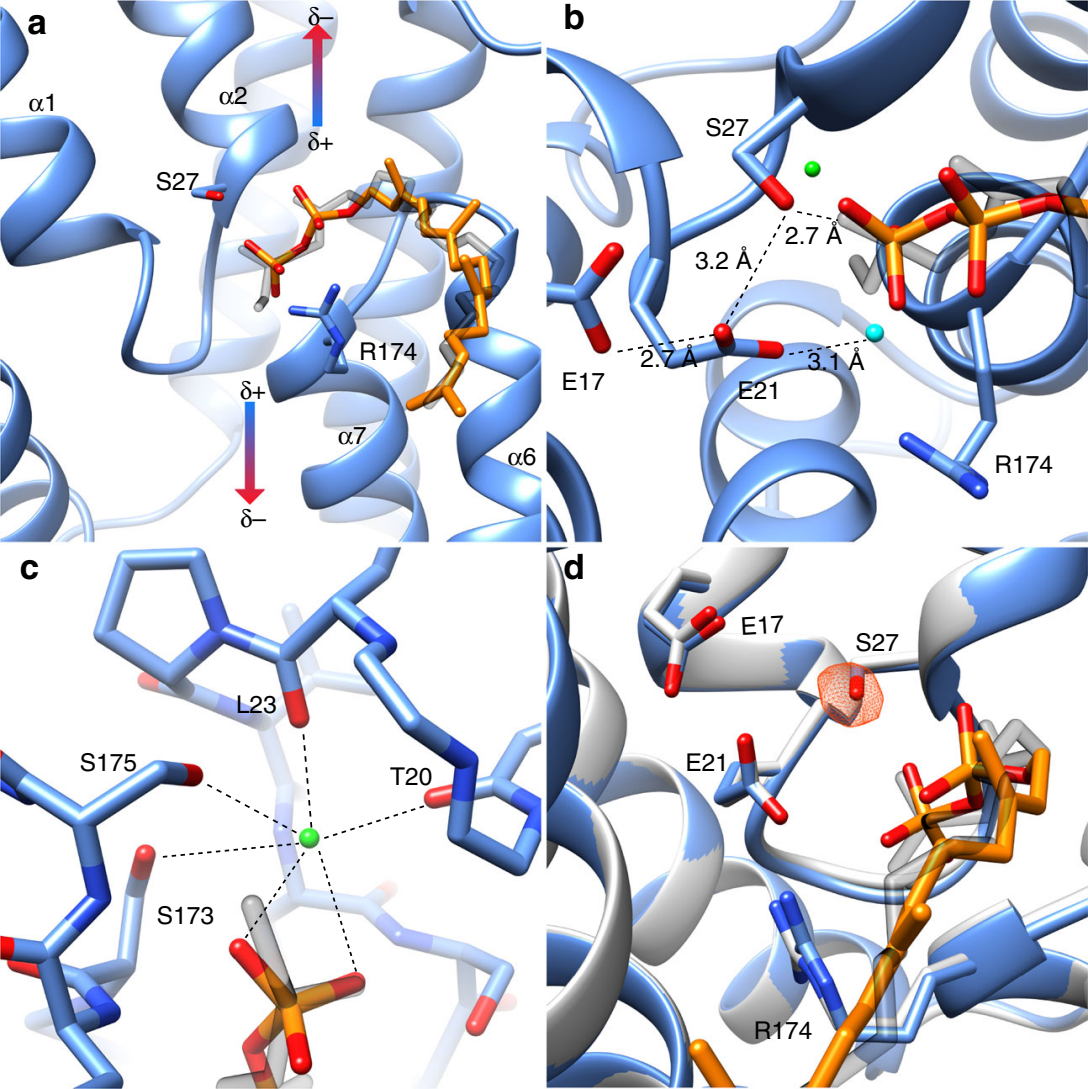
Modeling of C55-PP in the *Ec*UppP active site **a**. Overlay of the C55-PP pyrophosphate on the observed monoolein headgroup illustrates the coordination of the pyrophosphate by the N termini of α2 and α7. **b** Overlay of the C55-PP on the observed monoolein headgroup highlighting the interaction network of the key catalytic residues Ser27 (nucleophile), Glu21 (base), Glu17 (carboxyl–carboxylate pair), Arg174 (coordinates beta-phosphate). **c** Observed coordination sphere of a bound water we propose may represent a potential binding site for the requisite catalytic cation. The overlaid pyrophosphate would contribute two ligands and a favorable electronegative stabilization to the bound metal. **d** Overlay of wild-type (blue) and S27A mutant (gray) crystal structures show that the active site architecture is not perturbed by the S27A mutation. To confirm the identity the S27A mutant, the wild-type model was refined against the S27A data and an mF_o_-DF_c_ map was calculated to show the negative difference peak for the Ser27 side chain hydroxyl (contoured at 3σ)

We propose the active site bound monoolein lipid we observe is acting as a C55-PP mimic. While monoolein lacks the negative charge associated with the native substrate pyrophosphate moiety, like C55-PP it has a hydrophilic headgroup and single hydrophobic tail. Overlay of the monoolein headgroup with a pyrophosphate molecule allows us to make inferences about potential interactions, illustrating that UppP utilizes the pseudosymmetry of the reentrant helices to coordinate the two phosphate moieties of the pyrophosphate headgroup at the N terminus of the adjacent C-terminal reentrant helices α2 and α7 (Fig. 4a). The binding orientation is consistent with both a direct interaction with helix backbone amides, as suggested for binding of the Cl^−^ anion to the ClC transporter^35^, or via an electrostatic interaction with the potentially significant accumulated positive dipole moment at the N termini of helices^36^, here effectively doubled in impact by the disposition of the two inverted C-terminal reentrant helices buried in the middle of a membrane bilayer. Further electrostatic stabilization is realized by the Arg174 side chain guanadinium with the pyrophosphate moiety as well as weaker secondary interactions coming from hydrogen bonds with Glu21 and Thr28 (Fig. 4b). The UppP phosphatase activity is metal dependent with a strong preference for the divalent cations magnesium and calcium, the latter giving optimal activity in vitro^12, 13^. Density modeled as a water molecule is observed directly adjacent to the neutral monoolein headgroup and coordinated by hydrogen bonds with the primary and secondary alcohols of the glycerol moiety, as well as the main chain carbonyl oxygens of Leu23 and Thr20, and the side chain hydroxyls of the essential Ser173 and Ser175 (Fig. 4b–c). The hexadentate coordination number, ligand atom type, and relative disposition suggest this could represent the putative cation-binding site and the docked pyrophosphate positions the terminal electronegative phosphate for a direct bidentate interaction with the putative cation, providing an apical and equatorial ligand as well as the only complementary electronegative charge in an otherwise electrostatically neutral coordination sphere (the lack of the analogous geometry and charge in the bound monoolein headgroup is likely the reason we have captured a water rather than a well occupied magnesium or calcium ion in our experimental structures). Indeed, it is known that pyrophosphate-containing substrates in other enzyme systems serve to deliver bound catalytic magnesium or calcium ions to the active site^37^. Initial attempts to obtain C55-PP substrate or product complexes for both the native and a catalytic Ser27Ala mutant have been unsuccessful so far, and further experiments are required to confirm the substrate-binding mechanism and metal-binding site.

Based on our observations, we propose a catalytic mechanism for the phosphatase activity of UppP (Supplementary Fig. 7) in which Ser27 carries out a nucleophilic attack on the terminal phosphate of C55-PP, with the adjacent putative metal ion and Arg174 acting to coordinate, polarize, and stabilize the electrophilic phospho center and subsequent pentavalent transition state; the nearby Glu21 carboxylate (hydrogen bond of 3.1 Å with the Ser27 side chain hydroxyl) is well positioned for a role as the activating general base (Fig. 4b). The close proximity of the Glu17 and Glu21 side chains (2.8 Å) further suggests a potential role for a carboxyl–carboxylate interaction in modulating the pKa of Glu21 favorably for its general base role^38^. This facilitated attack of Ser27 results in the formation of a covalent phosphoserine intermediate that is subsequently hydrolyzed by an adjacent water to generate the C55-P and Pi reaction products and final regeneration of apo enzyme. A water molecule in our experimental maps is well positioned for this role, with again, general base assistance from the Glu21 carboxylate, its nearest hydrogen-bonding partner (Fig. 4b). Kinetic analysis of our wild-type, S27A and H30A mutant forms (Supplementary Fig. 1a) supports our mechanistic scheme, as does the nearly identical overlap of the native and S27A mutant structures showing the kinetic effects we observe arise primarily due to the catalytic role of this absolutely conserved residue (Fig. 4d).

A search for structural similarity using DALI^39^ failed to find any close structural homologs although remarkably the structure is similar to a previous prediction using co-evolution analysis coupled to Rosetta structure prediction^40^. Surprisingly, similarities to numerous proteins involved in the cross-membrane transport of small molecules were identified. Notably, similarity was detected to a ZIP zinc transporter^41^ (Top hit, DALI Z score 4.1, RMSD 4.1 Å across 139 residues, PDB 5TSA); a major facilitator superfamily (MFS) member multidrug transporter MdfA^42^ (DALI Z score 4.1, RMSD 6.0 Å across 140 residues, PDB 4ZP2); the eukaryotic chloride ClC transporter^43^ (DALI Z score 3.6, RMSD 5.4 Å across 88 residues, PDB 3ORG); and the sodium-dependent citrate symporter CitS^44^ (DALI Z score 3.5, RMSD 4.9 Å across 148 residues, PDB 5A1S). Comparison of these identified recurrent structural features including pseudosymmetry, inverted repeat topologies and, for the ClC transporter and CitS, the presence of reentrant helical/loop regions used to coordinate their anionic substrates similar to that observed here for *Ec*UppP. For CitS^44, 45^ and the MFS family of transporters^29, 46^, structures have been solved in discrete functional states highlighting the cycling between conformations open to opposing sides of the membrane to mediate transport. Indeed, this “alternating access” functionality is especially common in membrane proteins with interlocked inverted repeats, which exploit the pseudosymmetric arrangement to switch between conformations open to respective sides of the membrane^27^. Thus, the observed UppP topology—an interlocked inverted repeat with pseudosymmetry relating reentrant helical repeats—raises the intriguing possibility that UppP not only functions as a C55-PP phosphatase, but concomitantly plays a role in the recycling of C55-P back into the bacterial cytoplasm; the identity of such a C55-P flippase remains elusive.

Several glycolipid flippases involved in the synthesis of the bacterial cell wall or outer membrane have been identified including TagGH for teichoic acids^3^ and MsbA for lipopolysaccharide^47^. Notably, the identity of the lipid II flippase during peptidoglycan synthesis has been a matter of debate^48^ with recent structural and experimental evidence supporting a role of MurJ as a lipid II flippase^2, 18^. MurJ and UppP act on similar substrates (C55-PP-disaccharide–pentapeptide and C55-PP, respectively) and comparison of their structures highlights several related features. MurJ consists of 14 transmembrane helices with α1–α6 and α7–α12 related by (distorted) pseudosymmetry. The two lobes create a large cytoplasmic facing cavity with an exterior hydrophobic groove leading to a portal connecting to a strongly electropositive “proximal” site and weakly anionic “distal” site proposed to be the binding sites for the lipid II C55 tail, pyrophosphate, and disaccharide–pentapeptide, respectively. The portal and proximal site are similar in characteristics to the hydrophobic cleft and electropositive basin observed in UppP. The UppP structure also reveals a hydrophobic pocket situated below the active site cleft entrance and formed by amphipathic helix α3b, which is oriented parallel to the membrane plane and located at the cytoplasmic interface of the inner leaflet (Supplementary Fig. 3c). The ordered tails of two monoolein lipids are observed in the pocket, suggesting it may act as a binding site for the hydrophobic tail of the C55-PP substrate (Supplementary Fig. 3c). These shared structural characteristics of the substrate-binding sites are in agreement with the related lipid substrates of UppP and MurJ and provide further support for the proposed C55-PP-binding mechanism to UppP. Whether this comparison can be further extended to a related lipid flippase function for UppP, as its structural characteristics intriguingly suggest, requires further investigation.

In summary, we have solved the structure of *E. coli* UppP at 2.0 Å resolution revealing an unexpected inverted topology repeat similar to many cross-membrane transporters and indicating the basis for phosphatase action deep within the mid-layer of a bacterial membrane. Our results provide an important foundation on which to begin to further probe and understand the structural and functional mechanisms of this potential class of enzyme transporter and the design of antimicrobials that targets its essential role in virulence. Additionally, what remains unclear is how C55-PP generated de novo in the cytoplasm by the pathway terminating at UppS is dephosphorylated in its final necessary stage for subsequent use as a lipid carrier. Would, for example, a lipid II flippase such as MurJ, which operates in the opposite direction, promiscuously serve to flip C55-PP to the periplasmic space for subsequent phosphatase and recycling action by UppP as previously suggested^13^? Or is there a possibility UppP could access C55-PP substrate from both faces at its internalized mid-layer active site? These exciting and fundamental questions on bacterial cell wall biogenesis and lipid recycling are now made possible by the structural foundation provided here.

## Methods

### UppP purification

The gene encoding UppP from *Escherichia coli* K-12 (ATCC 10798) was cloned into a pET28a vector encoding an N-terminal hexahistidine tag and a thrombin cleavage site. UppP was overexpressed in *E. coli* C41 cells (Sigma), grown in ZYP-5052 autoinduction media at 37 °C for 3 h before lowering the temperature to 27 °C and allowing growth to continue overnight. Cells were harvested by centrifugation and resuspended in 20 mM HEPES pH 7.5, 500 m NaCl, 10% glycerol. Resuspended cells were lysed 2× using an EmulsiFlex-C5 homogenizer (Avestin). Cellular debris was pelleted by centrifugation at 15,000 × *g* for 0.5 h and membranes were pelleted by centrifuging the resultant supernatant at 200,000 × *g* for 1.0 h. UppP was solubilized in 20 mM HEPES pH 7.5, 500 mM NaCl, 10% glycerol, and 1% *N-*dodecyl-β-d-maltopyranoside (w/v) (DDM, Anatrace) for 1.0 h and centrifuged to remove insoluble material. The supernatant was loaded onto a 5 mL Ni-NTA Superflow (Qiagen) column pre-equilibrated with 20 mM HEPES pH 7.5, 500 mM NaCl, 30 mM imidazole, 0.016% DDM, washed with 60 mM imidazole, and UppP was eluted with 250 mM imidazole. The purified protein was desalted into 20 mM HEPES pH 8.0, 150 mM NaCl, 0.016% DDM, and the hexahistidine tag was removed by thrombin cleavage overnight. UppP was further purified by gel filtration using a Superdex 200 Increase 10/300 GL column equilibrated with 20 mM HEPES pH 7.0, 150 mM NaCl, 0.016% DDM before being concentrated to 12 mg mL^−1^ in an Amicon Ultra-15 concentrator (Millipore) with a molecular weight cutoff of 50 kDa.

### Crystallization

Lipid cubic phase (LCP) crystallization trials were performed by mixing UppP with monoolein (Sigma) in a 2:3 protein to lipid ratio (v/v). Crystallization trials were set up in 96-well glass Laminex sandwich plates (Molecular Dimensions) using an LCP Gryphon (Art Robbins Instruments). Aliquots of 100 nL LCP droplets were overlaid with 1 μL crystallization solutions and incubated at 20 °C. Initial crystals were obtained using a solution of 40% PEG 200, 100 mM NaCl, 100 mM LiSO_4_, and 100 NaCitrate pH 5. Optimized crystals grew in a solution containing 45–50% PEG 200, 0–150 mM NaCl, 200–400 mM LiSO_4_, and 100 mM NaCitrate pH 4. Crystals were harvested using MicroMounts (MiTeGen) and were frozen directly in liquid nitrogen. Selenomethionine-substituted protein crystallized with the addition of 2 mM β-mercaptoethanol throughout purification; however, the diffraction was of insufficient quality to permit structure solution. Mercury derivatized crystals were obtained using the same technique as above, but the concentrated UppP was incubated with a sixfold molar excess of ethyl mercury phosphate at 4 °C for 30 min before preparation of LCP, and the final crystals were obtained in a solution containing 45% PEG 200, 150 mM MgCl_2_, 400 mM LiSO_4_, 100 mM NaCitrate pH 5.

### Data collection and structure determination

X-ray diffraction data sets for both the native and mercury derivatized UppP crystals were obtained using Beamline 08ID-1 at the Canadian Light Source (CLS) in Saskatoon, Saskatchewan. Data were indexed and scaled using DIALS^49^, with a CC1/2 value of 0.998 overall and 0.419 in the highest resolution shell. Experimental phases were determined by autoSHARP^50^ using SHELXD^51^ for heavy atom substructure search, which identified a single mercury site at Cys165. Iterative cycles of model-building and refinement were performed with Coot^52^ and Phenix^53^. Data collection and refinement statistics are given in Table 1. The final model has good stereochemistry (98.9% Ramachandran favored, 0% outliers), few clashes (clashscore of 5.43), and a MolProbity score of 1.29.

Conservation analysis was carried out using Consurf^54^. Cleft analysis was carried out using the 3V channel finder^30^. Figures were created with chimera^55^ and PyMOL^56^.

### Kinetic assays

UppP phosphatase activity was measured using the EnzChek Phosphate Assay Kit (Thermo Fisher). The enzymatic assay reaction mixture contained 50 mM HEPES pH 7.0, 150 mM NaCl, 1 mM CaCl_2_, and 0.016% DDM. The enzymatic activity of wild-type UppP was measured using 0.9–24 μM farnesyl pyrophosphate (C15-PP) with 25 nM UppP. For comparison of wild-type UppP to S27A and H30A mutants, steady-state activity was measured in the presence of 200 μM C15-PP with 25 nM wild-type UppP, 1000 nM S27A UppP, and 100 nM H30A UppP. In the presence of the coupled assay enzyme purine nucleoside phosphorylase (PNP), the release of phosphate results in the conversion of 2-amino-6-mercapto-7-methylpurine riboside (MESG) to ribose 1-phosphate and 2-amino-6-mercapto-7-methylpurine, production of which can be measured at 360 nM. All assays were performed in triplicate. Initial velocity data were fitted to the Michaelis–Menten equation using Origin data analysis software.

### Crosslinking

Purified UppP was incubated at room temperature for 30 min with or without a 50-fold molar excess disuccinimidyl suberate (DSS) or ethylene glycol bis(succinimidyl succinate) (EGS). Tris(hydroxymethyl)aminomethane (Tris) was added to a final concentration of 50 mM to quench the crosslinking reaction. After incubating for a further 30 min, 10 μg samples of each reaction were loaded on a 12% SDS-PAGE gel for analysis.

### Data availability

Data supporting the findings of this manuscript are available from the corresponding author on reasonable request. Structure factors and atomic coordinates for the refined model have been deposited in the protein data bank with accession code 6CB2.

## Electronic supplementary material

Supplementary Information(PDF 13174 kb)
