# Supplementary material for: Crystal structure of an intramembranal phosphatase central to bacterial cell-wall peptidoglycan biosynthesis and lipid recycling
Source: Nat Commun. 2018 Mar 20;9:1159. doi: 10.1038/s41467-018-03547-8 (PMC5861054; doi:10.1038/s41467-018-03547-8)
Supplement: Supplementary file 1 — Supplementary Information(PDF 13174 kb) [file 41467_2018_3547_MOESM1_ESM.pdf]

Supplementary Information

Crystal structure of an intramembranal phosphatase central to bacterial cell-wall peptidoglycan biosynthesis and lipid recycling

Workman *et al.*

a.

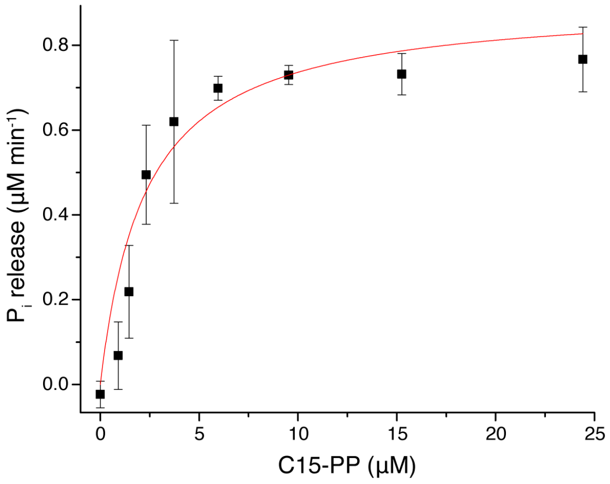

	This study	Chang *et al.*[12]	Manat *et al.*[13]
Wild-type	100%	100%	100%
S27A	0.020%	-	0.013%
H30A	27.0%	<1%	6.2%

b.

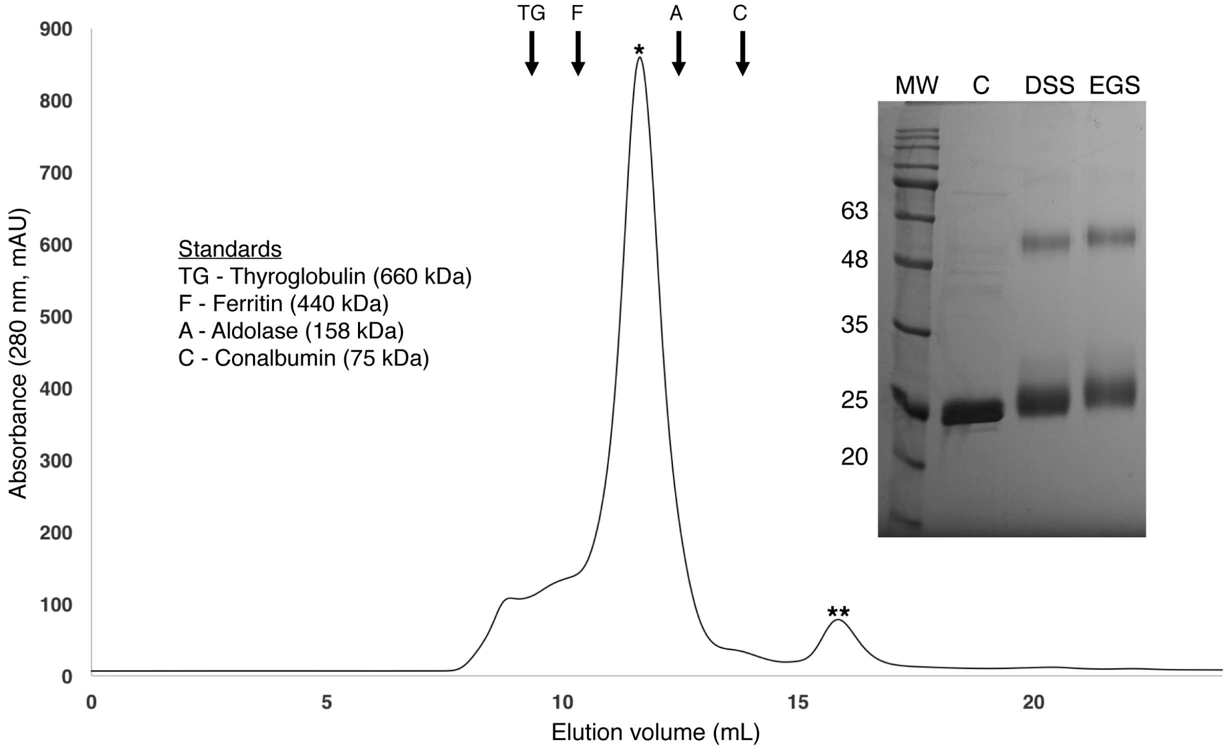

Supplementary Figure 1. Supporting biochemical evidence. a. Phosphatase activity of wild-type *Ec*UppP and comparison of wild-type activity to proposed catalytic mutants. The phosphatase assay was performed as described in Methods. Bars indicate one standard deviation from the mean absorbance measured in three replicates. b. Gel filtration chromatogram of Ni-NTA purified UppP using a Superdex 200 Increase 10/300 GL column. The elution volume of UppP (*) is consistent with the formation of a dimer (60 kDa) + two DDM micelles (175 kDa). The small peak (**) eluting after UppP corresponds to the thrombin used in removal of the hexahistidine affinity tag. Right inset shows SDS-PAGE analysis of UppP (C) incubated with disuccinimidyl suberate (DSS) or ethylene glycol bis(succinimidyl succinate) (EGS). Addition of either DSS or EGS results in the appearance of a single higher MW band at approximately 60 kDa, consistent with crosslinked UppP dimer.

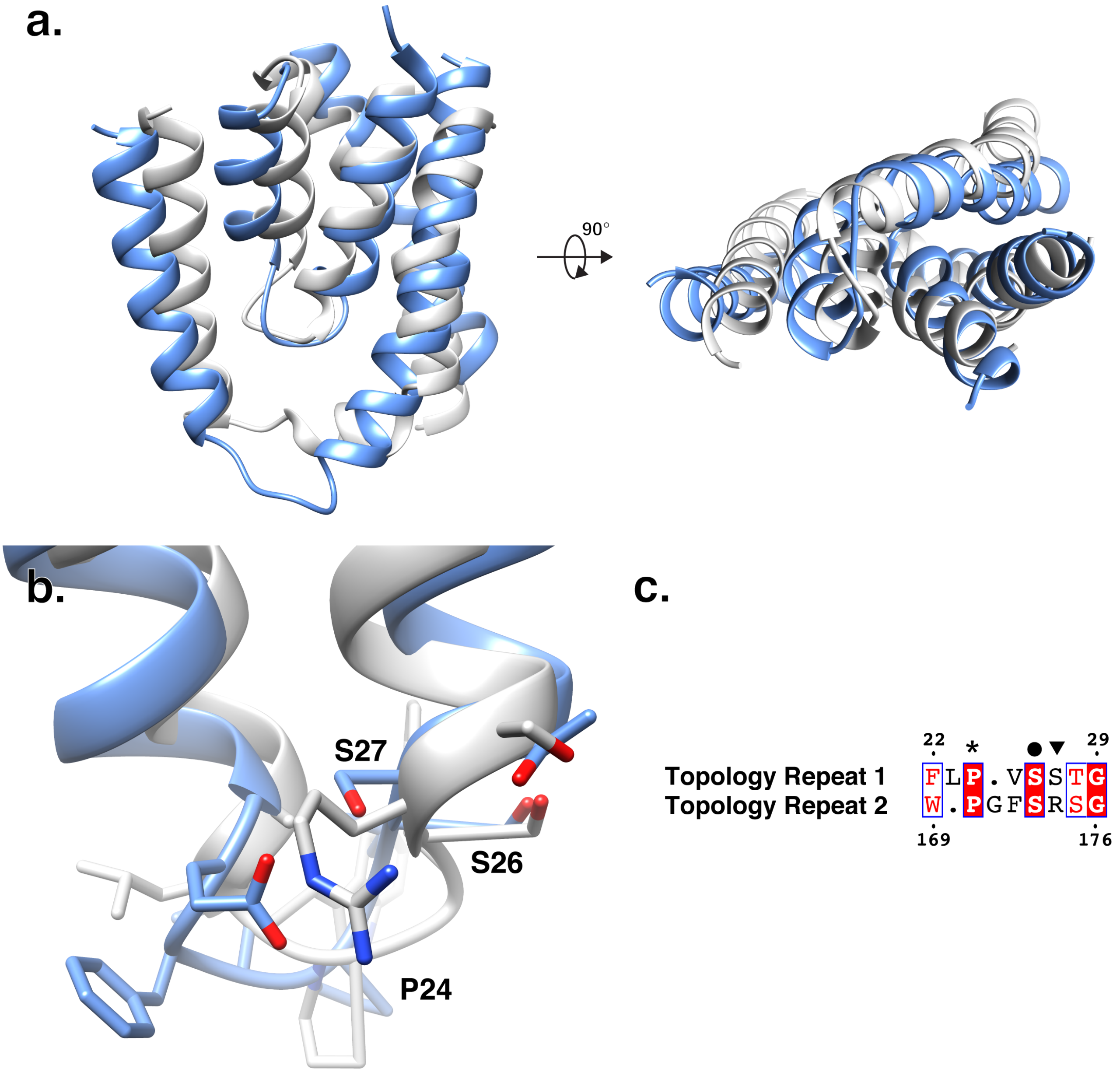

Supplementary Figure 2. Structural superposition of the inverted topology repeats. a. The first (blue) and second (grey) inverted topology repeats are shown structurally aligned from a side on and top down view. **b.** Superposition of just the reentrant helix-loop-helix motifs illustrating conservation of the key structural and catalytic residues. **c.** Sequence alignment of the reentrant loops indicating the crucial helix-breaking prolines (*), helix N-cap serines (●), and the direct alignment of the catalytic serine with the substrate binding arginine (▼).

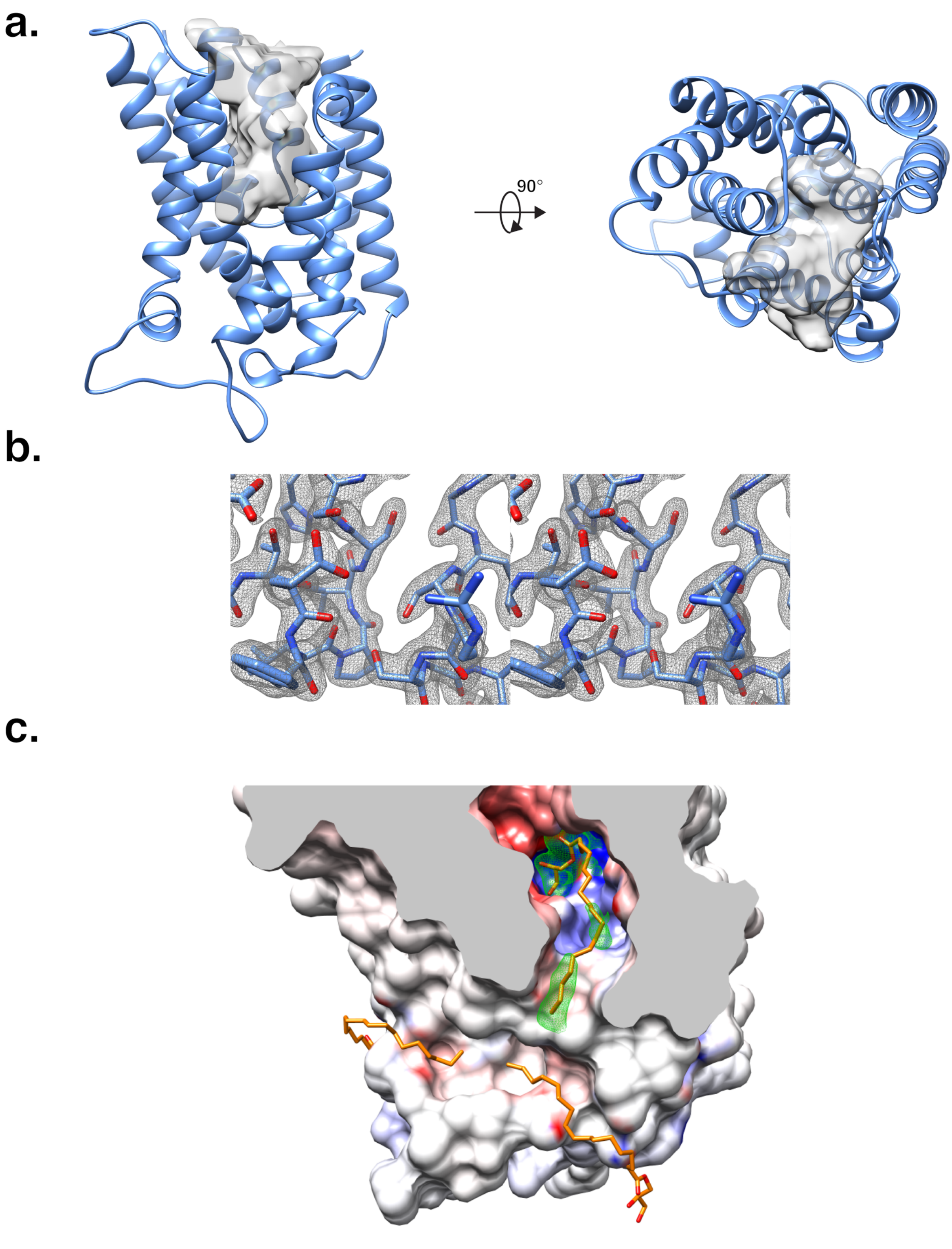

Supplementary Figure 3. The periplasmic oriented substrate binding cleft. a. The binding cleft volume generated by the 3V server is visualized as a transparent surface modeled on top of a cartoon representation of *Ec*UppP from a side on and top down view showing a deep cleft extending to the membrane midplane (volume 1914 Å^3). **b.** Stick model of conserved residues at the base of electropositive basin shown in stereo. 2mF_o-DF_c map density is shown contoured at 1.5σ **c.** mF_o-DF_c simulated annealing omit map for the substrate cleft bound monoolein lipid, contoured at 2σ. The polar and hydrophobic ends are clearly defined while the connecting acyl chain is less well ordered. The acyl chains of two additional monoolein molecules are observed in a hydrophobic pocket below the entrance to the active site cleft, revealing a potential binding site for the hydrophobic tail of C55-PP.

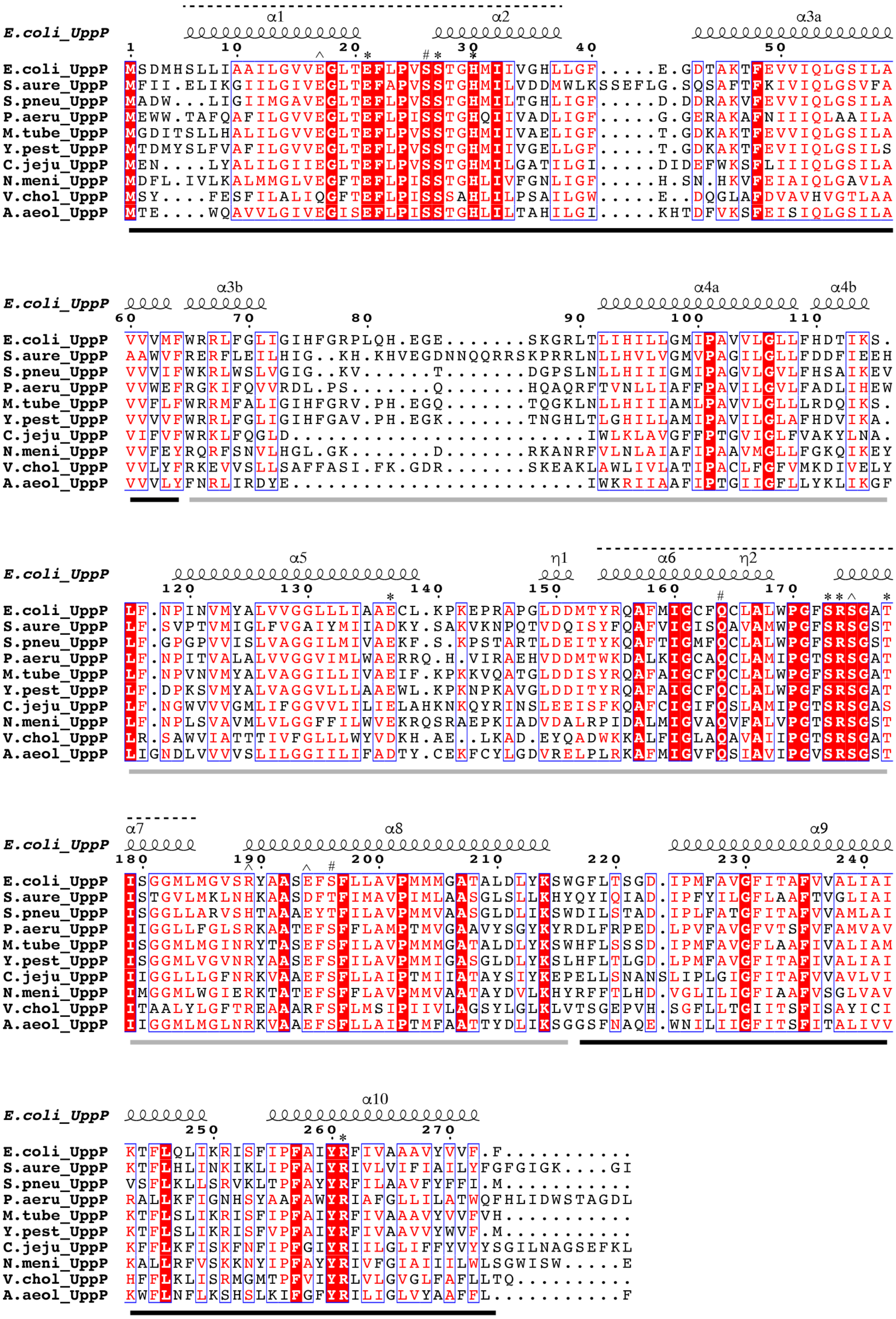

Supplementary Figure 4. Multiple sequence alignment of UppP orthologues. The amino acid sequence of *Escherichia coli* UppP (E.coli_UppP) is aligned with the following: *Staphylococcus aureus* (S.aure_UppP), *Streptococcus pneumoniae* (P.pneu_UppP), *Pseudomonas aeruginosa* (P.aeru_UppP), *Mycobacterium tuberculosis* (M.tube_UppP), *Yersinia pestis* (Y.pest_UppP), *Campylobacter jejuni* (C.jeju_UppP), *Neisseria meningitidis* (N.meni_UppP), *Vibrio cholerae* (V.chol_UppP), and *Aquifex aeolicus* (A._aeol_UppP). Dashed lines above α1-α2 and α6-α7 indicate the location of the two reentrant helices. Thick black or grey lines indicate that the sequence is part of either the first or second inverted topology repeat, respectively. Residual activity upon mutation to alanine is indicated by: * (≤10%), # (11-25%), ^ (26-50%).

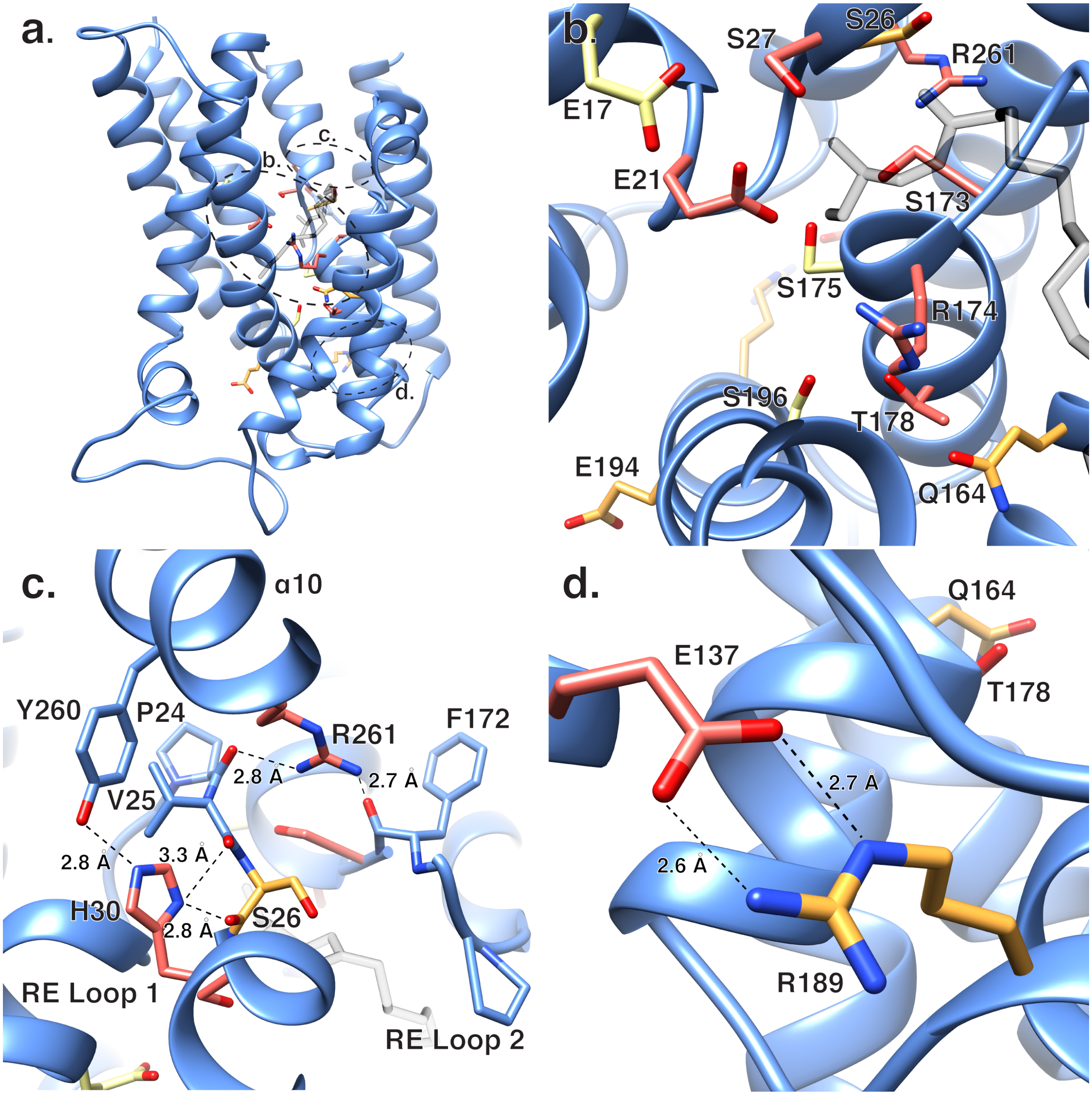

Supplementary Figure 5. Structural mapping of published activity mutants. a. Published activity mutants shown as stick and colored according to degree of effect from yellow (lowest) to pink (highest). Dotted circles highlight the zoomed regions in b-d.

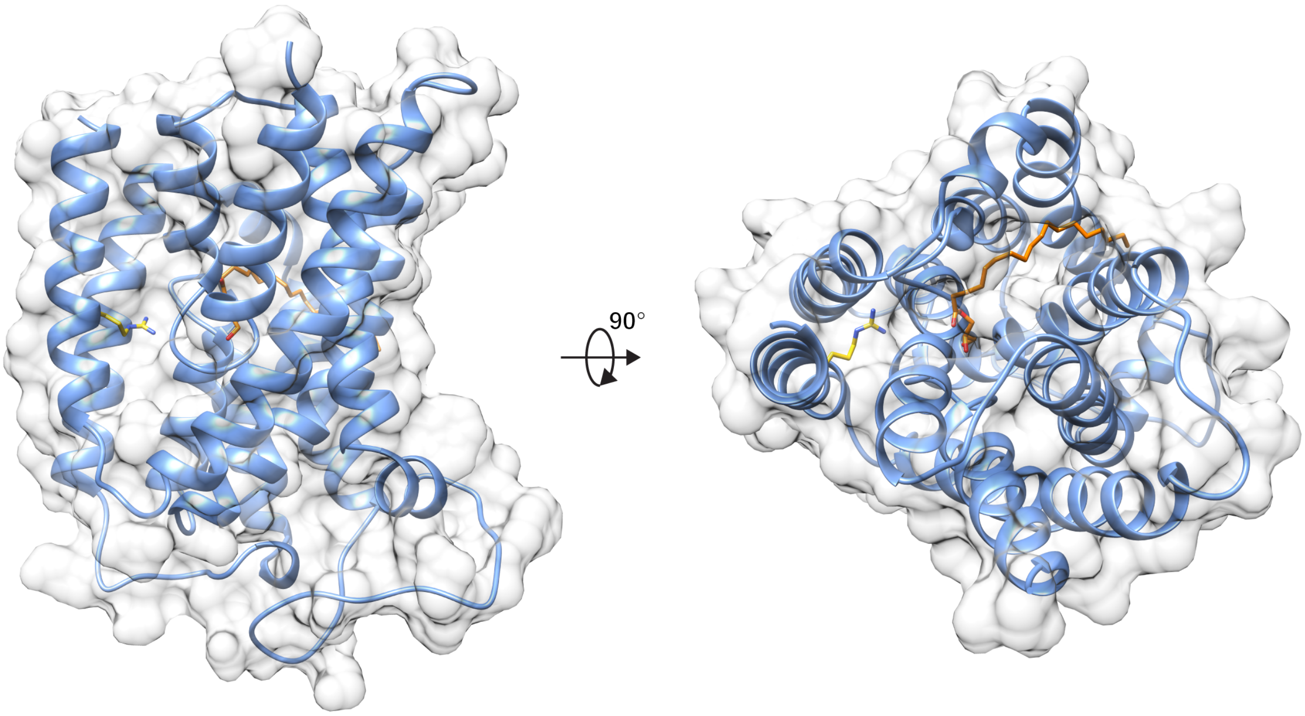

Supplementary Figure 6. Role of Arg261 in structural coordination of the active site architecture. Highly conserved Arg261 is buried in a hydrophobic interface at the membrane midplane and forms key interactions with the reentrant loops and helix α5 (see also Fig. 2d).

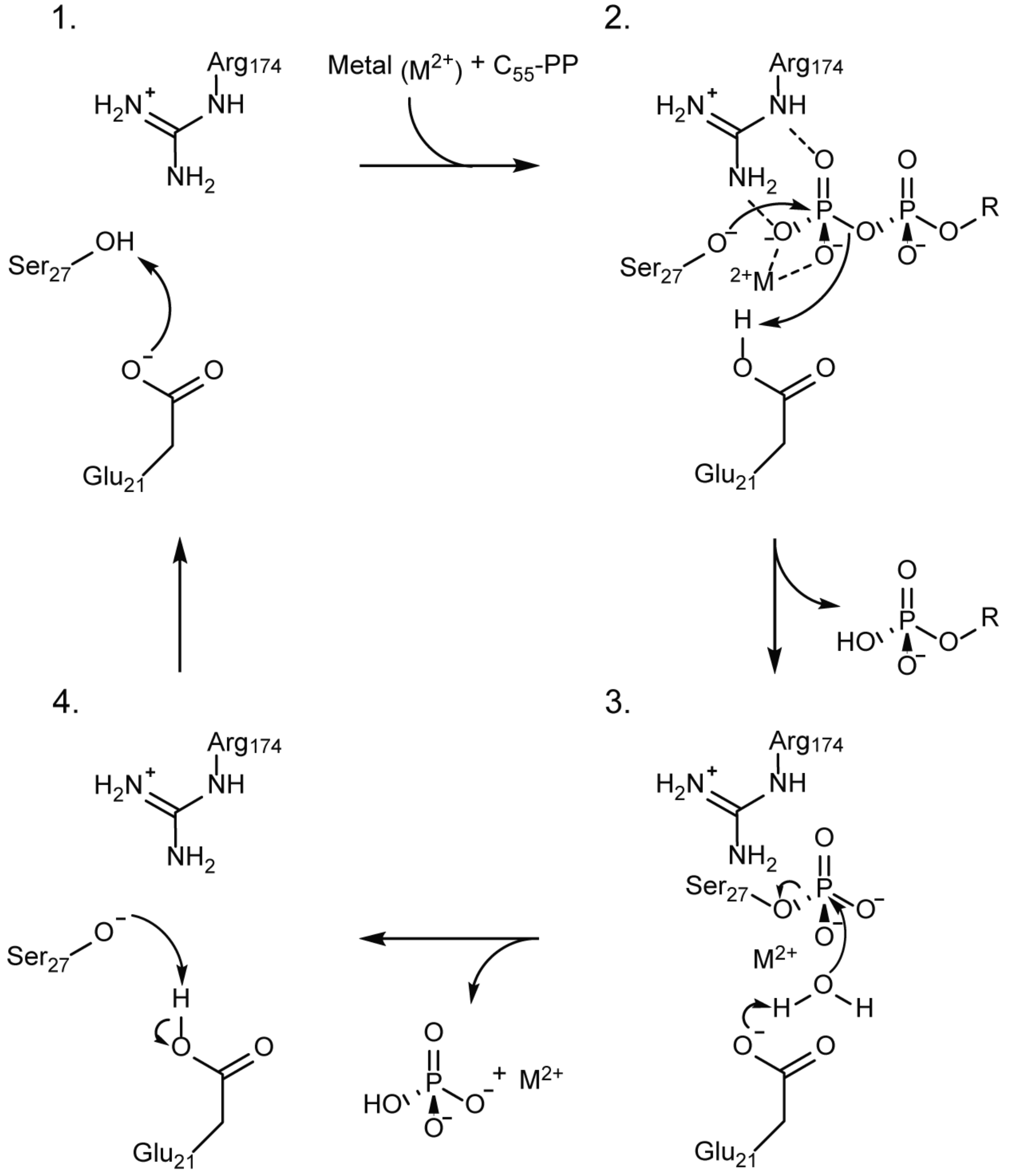

Supplementary Figure 7. Catalytic mechanism of *Ec*UppP phosphatase activity. 1. Activation of Ser27 by Glu21. 2. Ser27 carries out a nucleophilic attack on the terminal phosphate of C55-PP, stabilized by Arg174 and a putative divalent cation (M^{2+}), generating a phosphoenzyme intermediate, and releasing the C55-P product 3. A water is activated for attack by Glu21 to hydrolyze the phosphoenzyme intermediate. 4. Ser27 deprotonates Glu21 and returns the enzyme to its starting state.
